# Identification of a *Yarrowia lipolytica* acetamidase and its use as a yeast genetic marker

**DOI:** 10.1186/s12934-020-1292-9

**Published:** 2020-02-05

**Authors:** Maureen Hamilton, Andrew L. Consiglio, Kyle MacEwen, A. Joe Shaw, Vasiliki Tsakraklides

**Affiliations:** Novogy, Inc., 85 Bolton Street, Cambridge, MA 02140 USA

**Keywords:** *Yarrowia lipolytica*, Acetamidase, Acetamide, Fluoroacetamide, Dominant marker, Counterselectable marker, Marker removal, Marker recycling

## Abstract

**Background:**

*Yarrowia lipolytica* is an oleaginous yeast that can be genetically engineered to produce lipid and non-lipid biochemicals from a variety of feedstocks. Metabolic engineering of this organism usually requires genetic markers in order to select for modified cells. The potential to combine multiple genetic manipulations depends on the availability of multiple or recyclable selectable markers.

**Results:**

We found that *Y. lipolytica* has the ability to utilize acetamide as the sole nitrogen source suggesting that the genome contains an acetamidase gene. Two potential *Y. lipolytica* acetamidase gene candidates were identified by homology to the *A. nidulans* acetamidase *amdS.* These genes were deleted in the wild-type *Y. lipolytica* strain YB-392, and deletion strains were evaluated for acetamide utilization. One deletion strain was unable to grow on acetamide and a putative acetamidase gene *YlAMD1* was identified. Transformation of *YlAMD1* followed by selection on acetamide media and counterselection on fluoroacetamide media showed that *YlAMD1* can be used as a recyclable genetic marker in *Saccharomyces cerevisiae* and *Ylamd1Δ Y. lipolytica*.

**Conclusions:**

These findings add to our understanding of *Y. lipolytica* nitrogen utilization and expand the set of genetic tools available for engineering this organism, as well as *S. cerevisiae*.

## Background

*Yarrowia lipolytica* is a non‐conventional yeast of interest to the biotechnology industry that is rapidly emerging as a valuable host for the production of a variety of lipid and non-lipid biochemicals [1]. It is a dimorphic, oleaginous yeast that has been approved by the US Food and Drug Administration for use in manufacturing processes that are generally regarded as safe (GRAS) [2]. It possesses unique phenotypes, including hydrocarbon assimilation [3–8], specialty lipid and organic acid production [3, 9–13], and resistance to harsh environments including high salinity [3, 14], broad pH range [3, 15], and ionic liquids [3, 16].

*Yarrowia lipolytica’s* physiology, metabolism, and genetic regulation diverge significantly from more well‐studied and characterized yeasts such as *Saccharomyces cerevisiae* creating the need to adapt culture and genetic tools [17]. Metabolic engineering of yeast strains usually requires genetic markers in order to select for those cells that contain the desired genetic modifications. The construction of multiple successive genetic modifications is typically limited by the number of selection markers available in the host or requires marker recycling. There are two types of selection markers used for genetic manipulations in *Y. lipolytica* [18]: auxotrophic markers [19–21] and dominant markers [22–25]. Currently, only the uracil auxotrophy marker is counterselectable and lends itself to marker recycling through the use of 5′-fluoroorotic acid.

The *Aspergillus nidulans* acetamidase gene *amdS* has been successfully used as a dominant selection marker in filamentous fungi, the yeast *Kluyveromyces lactis*, and *S. cerevisiae* [26–32]. Acetamidase hydrolyzes acetamide to acetate and ammonia, making both carbon and nitrogen available to the cell and enabling growth on acetamide as the sole source of either of these elements [33, 34]. As a marker, *amdS* can be recycled through counterselection with fluoroacetamide, an acetamide homologue that is converted to the toxic compound fluoroacetate in the presence of active acetamidase [35, 36]. Fluoroacetamide counterselection has been applied to cure genetically engineered strains from recombinant constructs carrying the *amdS* gene [37].

In the present study, we examine acetamide utilization by *Y. lipolytica*, identify the gene responsible for the major *Y. lipolytica* acetamidase activity, and demonstrate its use as a counterselectable marker in this organism as well as *S. cerevisiae*.

## Results and discussion

### Acetamide utilization in *Y. lipolytica*

**Fig. 1:**
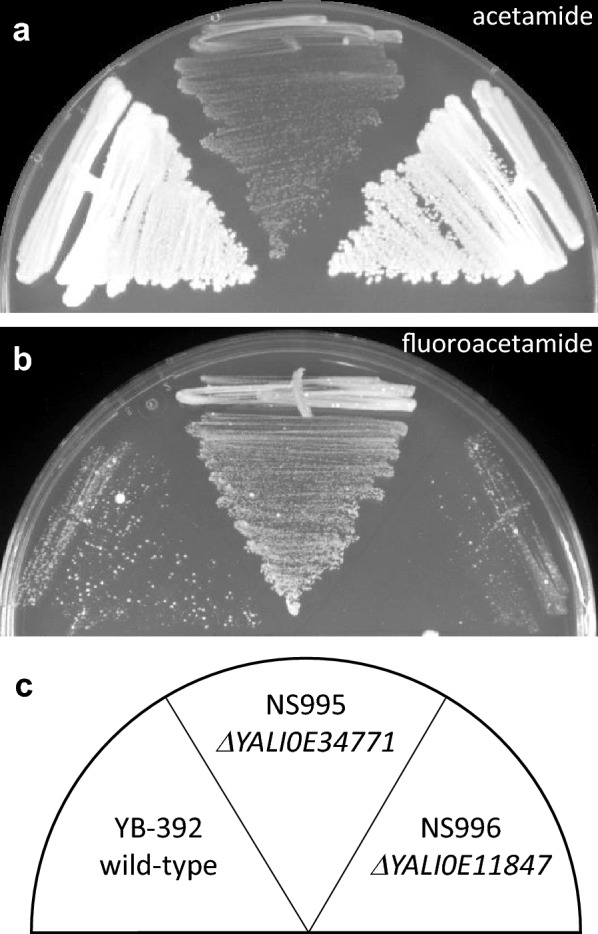
*Y. lipolytica* growth on acetamide and fluoroacetamide. Strains YB-392, NS995, and NS996 were streaked on defined media containing **a** 2.3 g/L acetamide as the sole nitrogen source, or **b** 5 g/L ammonium sulfate and 4.6 g/L fluoroacetamide. Plates were incubated at 30 °C for 5 days

To study acetamide utilization in *Y. lipolytica*, we tested growth of wild type strain YB-392 on defined media containing either acetamide or a combination of ammonium sulfate and fluoroacetamide as the sole nitrogen source. YB-392 was able to grow on acetamide media suggesting the presence of a functional acetamidase gene (Fig. 1a). When plated on fluoroacetamide media, YB-392 initially doesn’t grow (in agreement with the presence of a functional acetamidase) but gives rise to colonies after 5 days indicating it can bypass the toxicity of fluoroacetamide (Fig. 1b).

**Fig. 2:**
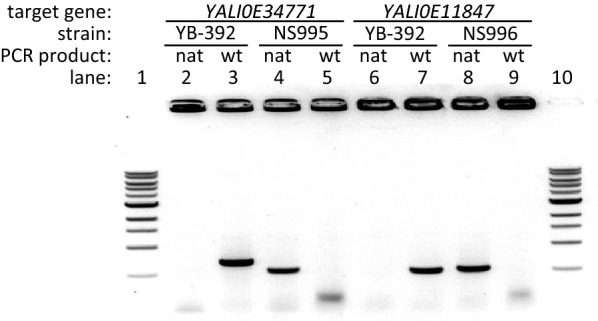
Gene deletion confirmation. Genomic DNA from strains YB-392, NS995 and NS996 was probed for the presence of the deletion target genes (“wt”) and the integrated antibiotic resistance cassette (“nat”) by PCR. YB-392 contains both the *YlAMD1* gene *YALI0E34771* (lane 3) and *YALI0E11847* (lane 7) but neither *nat1* integration-specific product (lanes 2 and 6). PCR confirms the absence of *YALI0E34771* and *YALI0E11847* in NS995 and NS996 respectively (lanes 5 and 9) while each of these strains contains the targeted-integration specific *nat1* product (lanes 4 and 8)

As there are no known acetamidase genes in *Y. lipolytica*, we searched for the gene(s) responsible for growth on acetamide. We used the *A. nidulans amdS* amino acid sequence to query the *Y. lipolytica* genome. *A. nidulans* is a filamentous fungus and its *amdS* gene was functional in the yeast *S. cerevisiae* that shares many selection markers with *Y. lipolytica*. Our BLAST [38] search identified the two closest *amdS* homologues: *YALI0E34771* (E score 1 × 10^−86^) and *YALI0E11847* (E score 3 × 10^−79^). To evaluate their role in acetamide utilization, we used targeted integration of the *nat1* marker to delete each of these genes, creating strains NS995 (*YALI0E34771::nat1*) and NS996 (*YALI0E11847::nat1*) (Table 1). To confirm the gene knock-outs and *nat1* insertions, genomic DNA was isolated from strains YB-392, NS995, and NS996 and screened by PCR as well as sequencing of the target loci in the modified strains. PCR results confirmed the replacement of each target gene by the antibiotic resistance marker (Fig. 2). Sequencing results also confirmed the presence of the *nat1* sequence in place of *YALI0E34771* and *YALI0E11847* in NS995 and NS996 respectively (data not shown).

**Table 1:** Strains used in this study

Strain	Genotype	Source
YB-392 *Y. lipolytica*	Wild type	NRRL#YB-392
S288C *S. cerevisiae*	*Mat*-*alpha Suc2 mal mel gal2 CUP1 flo1 flo8*-*1 hap1*	ATCC 204508 strain S288C
NS995 *Y. lipolytica*	*YALI0E34771::nat1*	This study
NS996 *Y. lipolytica*	*YALI0E11847::nat1*	This study

**Fig. 3:**
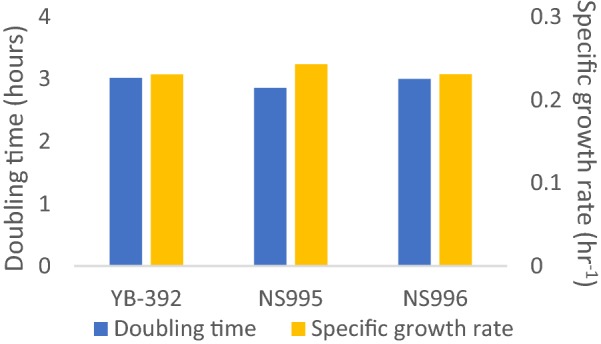
Growth comparison. 1 L batch fermentations of strains YB-392, NS995, and NS996 were sampled every 2 h. Specific growth rates and doubling times were determined using optical density readings (OD_600_)

Growth comparisons of *Y. lipolytica* strains YB-392, NS995, and NS996 were carried out in batch fermentations to determine whether the gene deletions made in NS995 and NS996 had a deleterious effect on growth rates. The 3 strains were grown in 1 L fermenters using ammonium sulfate as a nitrogen source. Samples were removed every 2 h and growth continued until nitrogen was exhausted. Doubling times and specific growth rates were calculated from the optical densities (OD_600_) (Fig. 3). Based on the fermentation data the deletion of *YALI0E34771* and *YALI0E11847* and their replacement with *nat1* did not affect the growth rate of either strain on ammonium when compared to the parental wild-type strain YB-392.

We tested the deletion strains for growth on acetamide and fluoroacetamide in a side by side comparison (Fig. 1). YB-392 and NS996 behaved similarly on each plate suggesting that *YALI0E11847* is likely not involved in acetamide utilization. In contrast, NS995 lost the ability to grow well on acetamide and gained resistance to fluoroacetamide. We conclude that *YALI0E34771* is most likely involved in acetamide utilization under the conditions tested in this study and refer to this gene as *YlAMD1*.

**Fig. 4:**
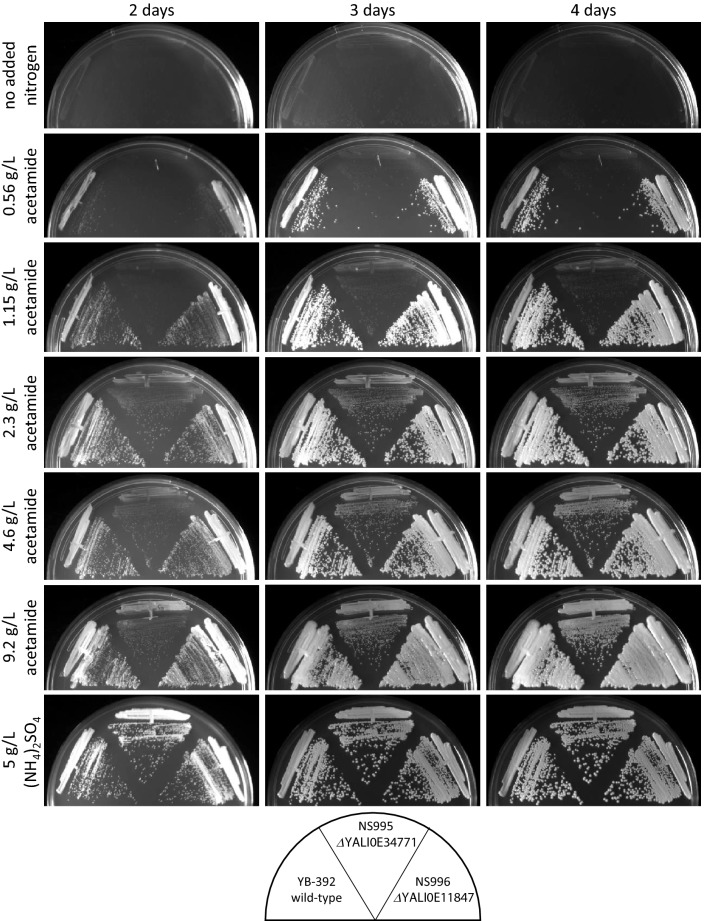
Acetamide titration. Strains YB-392, NS995, and NS996 were streaked on defined media containing no added nitrogen source, acetamide as the sole nitrogen source, or ammonium sulfate as the sole nitrogen source in the concentrations shown. Plates were incubated at 30 °C for 2–4 days

There is a small amount of NS995 growth on acetamide (Fig. 1a) suggesting that minor pathways of acetamide utilization could be present in addition to *YlAMD1* or that the strain is able to scavenge nitrogen from unknown impurities in the media. To investigate this growth further, we performed a titration of acetamide in defined media agar plates and followed the growth of YB-392, NS995 and NS996 over 4 days (Fig. 4). None of the strains formed colonies in the absence of an added nitrogen source indicating that other media components did not contain nitrogen accessible and sufficient for growth. All three strains showed robust growth when ammonium sulfate was supplied. For YB-392 and NS996, growth on 2.3 g/L or higher acetamide was similar to growth on ammonium sulfate whereas growth at lower acetamide concentrations required an additional day or two to form larger, defined colonies. N995 had a visible growth defect on acetamide compared to the other strains, in agreement with a role for *YlAMD1* in acetamide utilization. This *Ylamd1Δ* strain was able to form small slow-growing colonies at rates dependent on acetamide concentration. This growth could be due to a secondary acetamidase activity or an impurity in the acetamide supply.

### Evaluation of *YlAMD1* as a selectable and counterselectable marker in *Y. lipolytica* and *S. cerevisiae*

**Fig. 5:**
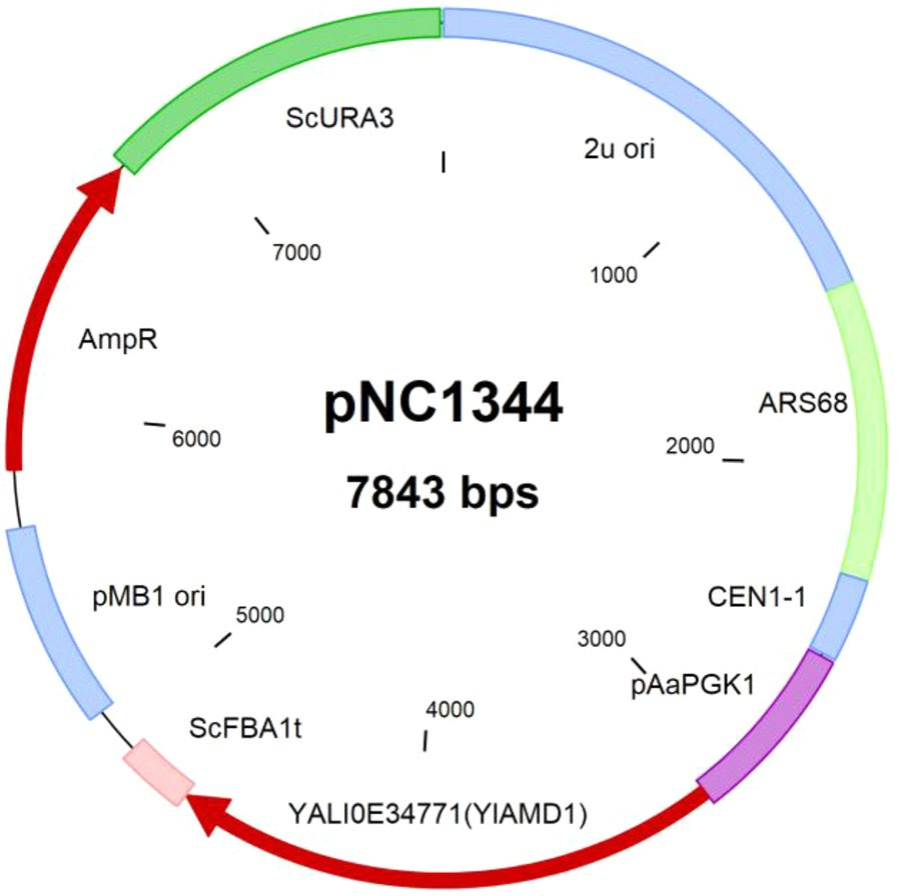
*YlAMD1* plasmid map. pNC1344 contains sequences for propagation in *E. coli*, *Y. lipolytica* and *S. cerevisiae* and has an expression cassette capable of expressing *YlAMD1* in both *Y. lipolytica* and *S. cerevisiae*

**Fig. 6:**
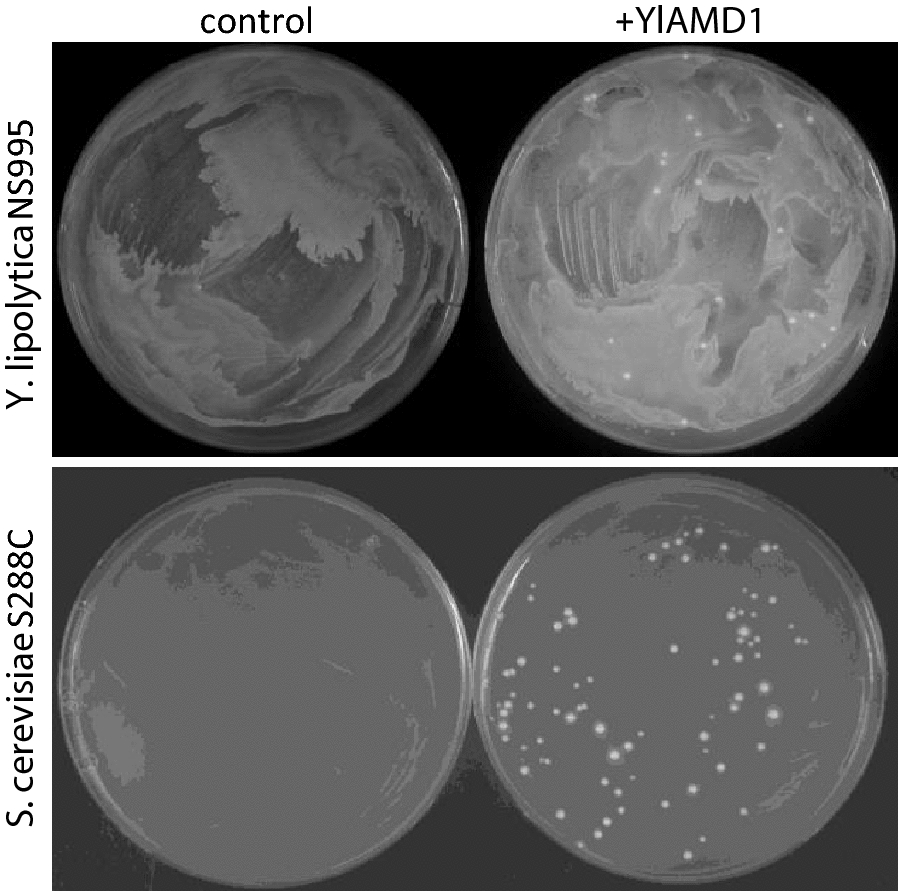
*YlAMD1* enables acetamide utilization and serves as a positive selection marker in *Y. lipolytica Ylamd1Δ* strain NS995 and *S. cerevisiae* S288C. Control (left) and pNC1344-*YlAMD1* (right) transformations were plated on 2.3 g/L acetamide plates and incubated at 30 °C for 5 days (*Y. lipolytica* NS995, top) or 2 days (*S. cerevisiae* S288C, bottom)

We evaluated *YlAMD1* as a selectable marker by transforming both *Y. lipolytica Ylamd1Δ* strain NS995 and haploid *S. cerevisiae* strain S288C with a plasmid capable of expressing *YlAMD1* in either organism (pNC1344, Fig. 5). Control transformations failed to form colonies while *YlAMD1*-transformed cells gave rise to well-defined colonies on acetamide plates (Fig. 6).

**Fig. 7:**
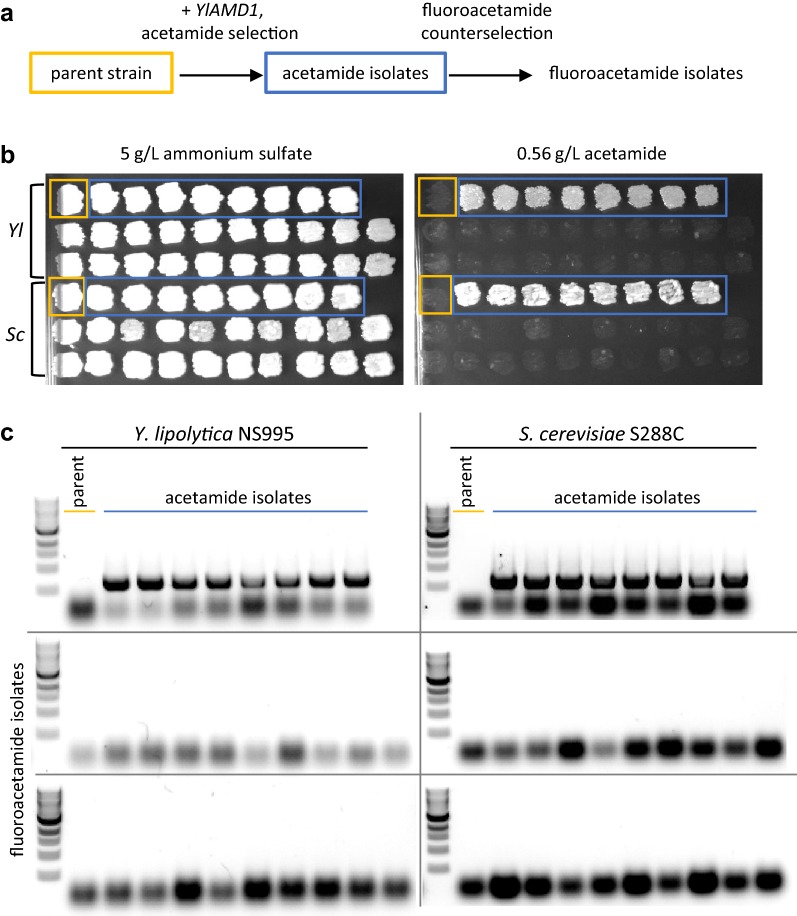
Plasmid curing through fluoroacetamide counterselection. **a** Acetamide-positive pNC1344-*YlAMD1* transformants were plated on fluoroacetamide media for counterselection of the acetamidase plasmid. **b** Parent strains *Y. lipolytica* NS995 (“Yl”) and *S. cerevisiae* S288C (“Sc”), acetamide-positive pNC1344-*YlAMD1* transformants and fluoroacetamide-resistant isolates derived from these transformants were patched on defined media containing either 5 g/L ammonium sulfate or 0.56 g/L acetamide as the sole nitrogen source and incubated at 30 °C for 2 days. **c** The strains in B were probed for the presence of the plasmid using primers specific for the *YlAMD1* gene

Acetamide-positive transformants from each strain background were used to investigate whether the *YlAMD1* plasmid can be cured using the acetamide homologue fluoroacetamide. These acetamide isolates were streaked on fluoroacetamide media for counterselection and gave rise to a few fluoroacetamide-resistant colonies (see flow diagram in Fig. 7a), suggesting loss of the *YlAMD1* plasmid. Fluoroacetamide-resistant *S. cerevisiae* colonies were restreaked on fluoroacetamide media to ensure loss of the multicopy 2μ plasmid. The parent strain (S288C or NS995), several *YlAMD1* transformants and their fluoroacetamide-resistant derivatives were patched onto defined media with either 5 g/L ammonium sulfate or 0.56 g/L acetamide as nitrogen source. Those isolates that arose from fluoroacetamide counterselection no longer grew on acetamide (Fig. 7b). PCR screening confirmed the presence of *YlAMD1* in the original pNC1344 transformants and showed *YlAMD1* was not present in the parental strains NS995 and S288C, or in the isolates after counterselection on fluoroacetamide plates (Fig. 7c). The lineages leading from parent strains to fluoroacetamide-resistant isolates are described in Additional file 1.

Taken together, these results demonstrate that *YlAMD1* can be used as a dominant and counterselectable marker for genetic modifications in *Ylamd1*Δ *Y. lipolytica* strains as well as *S. cerevisiae*, allowing for marker recycling for future bioengineering of the strains with the same marker. Although the *Ylamd1Δ Y. lipolytica* strain is an acetamide auxotroph, the deletion is not expected to affect central metabolism to the extent of other auxotrophic markers, such as *URA3* and *LEU2*. *YlAMD1* may therefore be particularly useful in physiology studies or in industrial applications were auxotrophic supplementation is undesirable [39].

## Conclusions

In the present study, we examined acetamide utilization by *Y. lipolytica* and determined that this oleaginous yeast is able to grow on acetamide as the sole nitrogen source. After deletion of two candidate genes, we showed that *YlAMD1* encodes the major *Y. lipolytica* acetamidase activity and demonstrated its use as a counterselectable marker in this organism and *S. cerevisiae*. These findings add to our understanding of *Y. lipolytica* metabolism and expand the set of genetic tools available for engineering this organism.

## Methods

### Strains, media, and cultivation methods

The wild type yeast strains used for this study were *Y. lipolytica* YB-392 from the ARS (NRRL) culture collection and *S. cerevisiae* S288C (ATCC 204508). Yeast strains were cultivated under non-selective conditions in either YPD media (10 g/L yeast extract, 20 g/L bacto peptone, 20 g/L glucose) or defined medium containing 20 g/L glucose, 5 g/L (NH_4_)_2_SO_4_, 3 g/L KH_2_PO_4_, 0.5 g/L MgSO_4_·7H_2_O, 0.05 mg/L biotin, 25 mg/L myo-inositol, 1 mg/L d-pantothenic acid, 1 mg/L nicotinic acid, 1 mg/L thiamine, 1 mg/L pyridoxine, 0.2 mg/L p-aminobenzoic acid, 1 mg/L H_3_BO_3_, 0.3 mg/L CuSO_4_·5H_2_O, 0.1 mg/L KI, 0.4 mg/L Na_2_MoO_4_·2H_2_O, 4.5 mg/L ZnSO_4_·7H_2_O, 3 mg/L FeSO_4_·7H_2_O, 1 mg/L MnCl_2_·2H_2_O, 15 mg/L EDTA, 0.3 mg/L CoCl_2_·6H_2_O, 4.5 mg/L CaCl_2_·2H_2_O. Acetamide selection media was prepared as for the defined media above except that 5 g/L (NH_4_)_2_SO_4_ was replaced with 0.56–9.2 g/L acetamide (Sigma-Aldrich, catalog #A0500–100 g) and with the addition of 6.6 g/L K_2_SO_4_ and 5.25 g/L disodium phthalate (buffers at pH 5.6–5.8) [40]. For negative selection on fluoroacetamide, 4.6 g/L fluoroacetamide (MP Biomedicals, LLC, catalog #213878) and 5.25 g/L disodium phthalate were added to defined media. For solid media 15 g/L of agar was added to the media described above. All yeast strains were cultivated at 30 °C. Antibiotic selection was achieved with the addition of nourseothricin at 50 μg/mL for *S. cerevisiae* and 500 μg/mL for *Y. lipolytica*.

### *YlAMD1* plasmid construction

Plasmid construction was based on standard molecular biology practices and protocols. Restriction enzymes and other molecular biology enzymes were acquired from New England Biolabs (Ipswich, MA). TOP10 electrocompetent (ThermoFisher Scientific, N.Y.) and DH5 alpha chemically competent (N.E. Biolabs) *E. coli* cells were used to propagate plasmids and the final construct, pNC1344, was confirmed by Sanger sequencing (Wyzer Biosciences). The *Y. lipolytica YALI0E34771* gene, *YlAMD1*, was PCR amplified from YB-392 genomic DNA with primer pair NP3157/NP3158, using Phusion DNA polymerase and cloned into an expression cassette using the *PGK1* promoter derived from *Arxula adeninivorans* and the *FBA1* terminator derived from *S. cerevisiae*. Both these elements drive gene expression in *Y. lipolytica* and *S. cerevisiae* (Novogy unpublished data). Additionally, pNC1344 carries ARS and CEN sequences for replication and plasmid maintenance in *Y. lipolytica* as well as the 2μ sequence for episomal plasmid maintenance in *S. cerevisiae*. The presence of the plasmid in pNC1344 transformants was confirmed using *YlAMD1* internal primers NP3379/NP3334. Primer sequences can be found in Additional file 1.

### Gene deletion in *Yarrowia lipolytica*

The *Y. lipolytica YALI0E34771* and *YALI0E11847* genes were deleted as follows: A two-fragment deletion cassette was amplified by PCR from a plasmid containing the nourseothricin resistance gene (*nat1)* such that the *nat1* gene was split into two fragments that overlapped and were flanked by ~ 50 bp of homology to the upstream and downstream regions of the coding sequence. The resulting PCR fragments were co-transformed into hydroxyurea-treated YB-392 [41]. Nourseothricin resistant colonies were screened by PCR to confirm the absence of the targeted gene and the presence of the *nat1* gene at the targeted locus. The primer pairs used to amplify the deletion cassette and probe for the presence of the target genes and targeted *nat1* integration are listed in Additional file 1. The modified loci in NS995 and NS996 were amplified with external primers and the fragments were sequenced to confirm gene replacement. PCR amplification and sequencing primers are listed in Additional file 1.

### Yeast transformations

For *Y. lipolytica* transformations, log phase cells were either treated with 50 mM hydroxyurea for 2 h (gene deletions) [41] or were processed directly for transformation (plasmid transformation) [23]. *Y. lipolytica* competent cells were prepared following a protocol adapted from Chen et al. [42]. Cells were washed with water and resuspended in a volume of water equal to the volume of the wet cell pellet. 50 µl was aliquoted per transformation reaction, cells were centrifuged, and the supernatant was discarded. 18 μL of DNA and 92 μL of transformation mix (80 μL 60% polyethylene glycol 4000, 5 µl 2 M dithiothreitol, 5 µL 2 M lithium acetate pH 6, 2 µl 10 mg/mL single-stranded salmon sperm DNA) were added to the cell pellet. The cells, DNA, and transformation mix were vortexed and then incubated at 39 °C for 1 h. For *S. cerevisiae* transformations, log phase cells were washed with water and resuspended in 1 mL water per 100 OD units of cells. The transformation protocol was adapted from Gietz and Woods [43]. 100 µl was aliquoted per transformation reaction. 14 μL of DNA and 286 μL of transformation mix (240 μL 50% polyethylene glycol 3350, 36 µl 1 M lithium acetate, 10 µl 10 mg/mL single-stranded salmon sperm DNA) were added to the cell pellet. The cells, DNA, and transformation mix were vortexed and then incubated at 42 °C for 40 min. For all yeast transformations, the cells were centrifuged after heat shock, the supernatant was discarded, and the cells were resuspended in 1 mL YPD and incubated overnight at 30 °C, 200 rpm. Following the overnight recovery, the transformations were plated to selective media and incubated at 30 °C.

## Bioreactor cultures for growth characterization

Frozen working-stocks of strains YB-392 and NS995 were spread onto a YPD plate and grown overnight at 30 °C. For each strain, a 10 µL loopful of cells was removed from the plate and used to inoculate a 250 mL Erlenmeyer flask with 50 mL of defined shake flask medium consisting of: glucose (30 g/L), (NH_4_)_2_SO_4_ (5 g/L), KH_2_PO_4_ (3 g/L), MgSO_4_·7H_2_O (0.5 g/L), d-biotin (0.05 mg/L), Ca-d-pantothenate (1 mg/L), nicotonic acid (1 mg/L), myo-inositol (25 mg/L), thiamine hydrochloride (1 mg/L), pyridoxal hydrochloride (1 mg/L), p-aminobenzoic acid (0.2 mg/L), Na_2_EDTA (1.5 mg/L), ZnSO_4_·7H_2_O (0.45 mg/L), MnCl_2_·2H_2_O (0.1 mg/L), CoCl_2_·6H_2_O (0.03 mg/L), CuSO_4_·5H_2_O (0.03 mg/L), Na_2_MoO_4_·2H_2_O (0.04 mg/L), CaCl_2_·2H_2_O (0.45 mg/L), (NH_4_)_2_FeSO_4_·6H_2_O (0.3 mg/L), H_3_BO_3_ (0.1 mg/L), KI (0.01 mg/L), potassium hydrogen phthalate (2 g/L), and disodium phthalate (12 g/L). The pH of the medium was 5.5. Inoculum flasks were cultured overnight at 30 °C with constant agitation of 200 rpm in a New Brunswick I26 incubator shaker, whereupon the OD_600_ was measured. The volume of each flask culture required to initiate its corresponding 1 L bioreactor at a T_0_ cell density of 1 OD_600_ was transferred to separate 50 mL sterile conical tubes. Each conical tube was then brought to 50 mL with sterile diH_2_O and centrifuged at 4000 rpm for 3 min in an Eppendorf 5810 R centrifuge. The supernatant was decanted and the cells were then resuspended in 50 mL sterile diH_2_O. This washed inoculum was used to inoculate two 1 L bioreactors (Dasgip 1.2 L vessels) with medium consisting of: glucose (40 g/L), (NH_4_)_2_SO_4_ (5 g/L), KH_2_PO_4_ (3 g/L), MgSO_4_·7H_2_O (0.5 g/L), D-biotin (0.05 mg/L), Ca-d-pantothenate (1 mg/L), nicotonic acid (1 mg/L), myo-inositol (25 mg/L), thiamine hydrochloride (1 mg/L), pyridoxal hydrochloride (1 mg/L), p-aminobenzoic acid (0.2 mg/L), Na_2_EDTA (1.5 mg/L), ZnSO_4_·7H_2_O (0.45 mg/L), MnCl_2_·2H_2_O (0.1 mg/L), CoCl_2_·6H_2_O (0.03 mg/L), CuSO_4_·5H_2_O (0.03 mg/L), Na_2_MoO_4_·2H_2_O (0.04 mg/L), CaCl_2_·2H_2_O (0.45 mg/L), (NH_4_)_2_FeSO_4_·6H_2_O (0.3 mg/L), H_3_BO_3_ (0.1 mg/L), and KI (0.01 mg/L). Process parameters included a pH control at 3.5 automatically adjusted with 10 N sodium hydroxide, a temperature of 30 °C, aeration at 1.0 vvm air, and agitation controlled at 1400 rpm. A sample of 5 mL was taken from each culture at 2-h intervals starting at ∆12 h with a Flownamics Seg-flow automated sampler and continuing until nitrogen was depleted. The samples were continuously held at 4 °C after each harvest. The OD_600_ was measured for each sample and used to calculate the doubling time and cell specific growth rate for each strain. This process was repeated, again with YB-392, and additionally with NS996. The results for both YB-392 cultures were averaged.

## Supplementary information

**Additional file 1.** Primers and primer sequences used in this study.

## Data Availability

All data generated or analysed during this study are included in this published article and its additional information files.
