# Supplementary material for: Identification of a Yarrowia lipolytica acetamidase and its use as a yeast genetic marker
Source: Microb Cell Fact. 2020 Feb 5;19:22. doi: 10.1186/s12934-020-1292-9 (PMC7003347; doi:10.1186/s12934-020-1292-9)
Supplement: Supplementary file 1 — Additional file 1. Primers and primer sequences used in this study. [file 12934_2020_1292_MOESM1_ESM.docx]

**Additional Table S1: Primers used for gene deletions**

| **Target Modification** | **5’ Fragment for deletion cassette** | | **3’ Fragment for deletion cassette** | | **Presence of target gene** | | **Presence of *nat1* integration** | |
| --- | --- | --- | --- | --- | --- | --- | --- | --- |
|  | Forward | Reverse | Forward | Reverse | Forward | Reverse | Forward | Reverse |
| *YALI0E34771::nat1* | NP2966 | NP2967 | NP2969 | NP2968 | NP2970 | NP2999 | NP2970 | NP2967 |
| *YALI0E11847::nat1* | NP2971 | NP2967 | NP2969 | NP2972 | NP2973 | NP3000 | NP2973 | NP2967 |

**Additional Table S2: Primers used for modified locus amplification and sequencing**

| **Target Modification** | **Amplification and sequencing primers** | |
| --- | --- | --- |
|  | Forward | Reverse |
| *YALI0E34771::nat1* | NP2970 | NP5412 |
| *YALI0E11847::nat1* | NP2973 | NP5414 |

**Additional Table S3: Primer Sequences**

| **Primer** | **Sequence** |
| --- | --- |
| NP2966 | CAACACTTACTACAATCTCAATCACCACCCTTATTTTGCAATGACCACTCTGGATGACAC |
| NP2967 | CCAGCACCTCGCTCTCGAGC |
| NP2968 | GACGAAAAGATCGTTGATAAATACACACATTTTTTCGTGTTTAAGGGCAGGGCATCGACA |
| NP2969 | GAGAGGACGGCGACCCTGAC |
| NP2970 | CAGCATGCAGTTCGAGACAG |
| NP2971 | GTCTCGGTGGGCTGATTCTGAGTATCGCAATATCACCAGCATGACCACTCTGGATGACAC |
| NP2972 | GAAAAGTTCCTGTTCAATAACATCAATTGTGGTCAAAATACTTAAGGGCAGGGCATCGAC |
| NP2973 | GCGAAGCTGTCTGACAAGTG |
| NP2999 | GACATGAGAAACTGAGGCGC |
| NP3000 | GCTCTTTGACGGAGAAAGG |
| NP3157 | GTGCTTTGTTTTTCAATTGGCAACCTATAACACAATGACCGACTACGAGGCCCGAAAAAC |
| NP3158 | CTCATTAAAAAACTATATCAATTAATTTGAATTAACTCACATAGCATGAGAGAACACCTC |
| NP3334 | GACCACCGTCGGGGAAGTAAAGAC |
| NP3379 | CCGACAACGCAGTACTGGTCAACG |
| NP5412 | GCACCAGAGGTGTGGTCTATC |
| NP5414 | CTGACTACAGTGCCGACTCTC |

**Additional Table S4: Plasmid Curing Lineages in Figure 7**

|  | 1 | 2 | 3 | 4 | 5 | 6 | 7 | 8 | 9 | 10 |
| --- | --- | --- | --- | --- | --- | --- | --- | --- | --- | --- |
| A | NS995 | NS995/pNC1344 #1 | NS995/pNC1344 #2 | NS995/pNC1344 #3 | NS995/pNC1344 #4 | NS995/pNC1344 #5 | NS995/pNC1344 #6 | NS995/pNC1344 #7 | NS995/pNC1344 #8 |  |
| B | 995 1-1 | 995 1-2 | 995 1-3 | 995 2-1 | 995 2-2 | 995 3-1 | 995 3-2 | 995 4-1 | 995 4-2 | 995 4-3 |
| C | 995 5-1 | 995 5-2 | 995 5-3 | 995 6-1 | 995 6-2 | 995 6-3 | 995 7-1 | 995 7-2 | 995 8-1 | 995 8-2 |
| D | NS567 | NS567/pNC1344 #1 | NS567/pNC1344 #2 | NS567/pNC1344 #3 | NS567/pNC1344 #4 | NS567/pNC1344 #5 | NS567/pNC1344 #6 | NS567/pNC1344 #7 | NS567/pNC1344 #8 |  |
| E | 567 1-1 | 567 1-2 | 567 1-3 | 567 2-1 | 567 2-2 | 567 2-3 | 567 3-1 | 567 3-2 | 567 4-1 | 567 4-2 |
| F | 567 5-1 | 567 5-2 | 567 6-1 | 567 6-2 | 567 6-3 | 567 7-1 | 567 7-2 | 567 8-1 | 567 8-2 | 567 8-3 |
